# Sexuality of Female Spina Bifida Patients: Predictors of a Satisfactory Sexual Function

**DOI:** 10.1055/s-0041-1732464

**Published:** 2021-07-27

**Authors:** Guilherme Lang Motta, Anna Bujons, Yesica Quiróz, Erika Llorens, Maira Zancan, Tiago Elias Rosito

**Affiliations:** 1 Surgery Department, Universidade Federal de Santa Maria, Santa Maria, Rio Grande do Sul, RS, Brazil; 2 Postgraduate Program in Health Sciences: Gynecology and Obstetrics, Universidade Federal do Rio Grande do Sul, RS, Brazil; 3 Pediatric Urology Department, Fundació Puigvert, Barcelona, Spain; 4 Gynecology and Obstetrics Department, Universidade Federal de Santa Maria, Santa Maria, Rio Grande do Sul, RS, Brazil; 5 Urology Department, Hospital de Clínicas de Porto Alegre, Porto Alegre, Rio Grande do Sul, RS, Brazil

**Keywords:** sexuality, spina bifida, sexual dysfunction, urinary incontinence, myelomeningocele, sexualidade, espinha bífida, disfunção sexual, incontinência urinaria, mielomeningocele

## Abstract

**Objective** To assess the sexual function of women with spina bifida (SB), and to verify the factors that influence their sexual function.

**Methods** A cross-sectional study in which a validated female-specific questionnaire was applied to 140 SB female patients from four different cities (Porto Alegre, Brazil; and Barcelona, Madrid, and Málaga, Spain) between 2019 and 2020. The questionnaires collected data on the clinical characteristics of SB, and female sexual function was assessed using the 6-item version of the Female Sexual Function Index (FSFI-6) validated to Portuguese and Spanish.

**Results** Half of the patients had had sexual activity at least once in the life, but most (57.1%) did not use any contraception method. Sexual dysfunction was present in most (84.3%) patients, and all sexual function domains were impaired compared those of non-neurogenic women. The presence of urinary and fecal incontinence significantly affected the quality of their sexual activity based on the FSFI-6.

**Conclusion** The specific clinical aspects of the SB patients, such as urinary and fecal incontinence, should be properly addressed by their doctors, since they are associated with reduced sexual activity and lower FSFI-6 scores in the overall or specific domains. There is also a need to improve gynecological care among sexually-active SB patients, since most do not use any contraceptive methods and are at risk of inadvertent pregnancy.

## Resumo

**Objetivo** Analisar a função sexual de pacientes do sexo feminino com espinha bífida (EB), e avaliar quais fatores influenciam na função sexual.

**Métodos** Uma pesquisa transversal em que um questionário validado para mulheres foi aplicado em 140 pacientes com EB de quatro cidades diferentes (Porto Alegre, Brasil; e Barcelona, Madri e Málaga, Espanha) entre 2019 e 2020. Os questionários coletaram dados sobre características clínicas da espinha bífida, e a função sexual feminina foi avaliada com a versão de seis itens do Índice de Funcionamento Sexual Feminino (IFSF-6) nas versões validadas para português e espanhol.

**Resultados** Metade das pacientes havia praticado atividade sexual pelo menos uma vez na vida, mas a maioria (57.1%) não utilizava nenhum método contraceptivo. A disfunção sexual estava presente na maioria das pacientes (84.3%), sendo todos os domínios de função sexual prejudicados em comparação com os de mulheres não neurogênicas. A presença de incontinência urinária e fecal afetou significativamente a qualidade da atividade sexual das pacientes.

**Conclusão** Aspectos clínicos específicos da EB, como incontinência urinária e fecal, devem ser adequadamente abordados pelos médicos assistentes, visto que estão associados à redução na atividade sexual e piores resultados no IFSF-6. Também é necessário melhorar o atendimento ginecológico das pacientes sexualmente ativas, uma vez que a maioria não utiliza métodos contraceptivos e corre o risco de gravidez inadvertida.

## Introduction

Spina bifida (SB) is the main neurological birth defect that occurs due to an impaired closure of the neural tube, leading to multi-systemic dysfunctions such as neurogenic bladder. 1 The life expectancy of SB patients has increased as a result of improved medical care; therefore, adult-life issues, such as social life and sexuality, have become growing concerns among this population. 1 2 There is consistent data associating the complications of SB, such as urinary incontinence (UI) and fecal incontinence, with negative effects on socialization. 1 3 Sexuality among SB patients is considered an important topic of discussion, and it lead to many studies on the male population. 4 5 Studies on female SB patients, however, are limited, and most have small sample sizes, are single-institution surveys, or use non-validated questionnaires. 6 7 8 9 These studies revealed that women with SB present higher sexual dysfunction rates than the general female population, and they suggest that some clinical factors, such as spinal-cord level and UI, could predict their sexual outcomes. The aim of the present study was to assess the sexual function of women with SB and to verify the factors that influence their sexual function.

## Methods

A cross-sectional study was implemented in four different SB centers (Spina Bifida associations inBarcelona, Madrid and Málaga, Spain; and the Urology Department at Hospital de Clínicas de Porto Alegre, in Porto Alegre, Brazil). Between 2019 and 2020, adult female SB patients who undergoing regular follow-up in the aforementioned centers were invited to participate in this study. Only women older than 18 years of age who could read and understand the questionnaire, after informed consent, were enrolled. The surveys were administered in person by trained interviewers who helped the patients to fulfill them. A non-probability purposive sampling of 210 patients was eligible and invited, with 140 accepting to participate after reading the informed consent (response rate of 66.6%).

The questionnaires collected data on demographics, socioeconomics, clinical and gynecological characteristics, and sexuality. Female sexual function was assessed using the 6-Item Version of the Female Sexual Function Index (FSFI-6) validated to Spanish and Portuguese. 10 11 12 It consists of a questionnaire that approaches the following sexual function domains: desire, arousal, lubrication, orgasm, satisfaction, and pain. Each item has a score varying from 0 to 5, whose sum provides the final score. A FSFI-6 total score ≤ 19 was considered a positive screening for female sexual dysfunction (FSD). 12 Sexual activity was defined as having a history of at least one sexual intercourse. The body mass index (BMI) was calculated using the patient's weight in kilograms divided by the square of height in meters, and obesity of was defined as a BMI score ≥ 30. Fecal incontinence or UI were defined as involuntary leakage of urine or feces. Psychological disorders were identified according to the patient's report, and they included the following conditions: depression, anxiety, or mood disorders.

The authors followed guidelines of the Strengthening the Reporting of Observational Studies in Epidemiology (STROBE) statement during the preparation oif the present manuscript. 13 The following statistical tests utilized were used: Chi-squared, Fisher exact, and Mann-Whitney, and they were analyzed using the Statistical Package for the Social Sciences (IBM SPSS Statistics for Windows, IBM Corp., Armonk, NY, US) software, version 25.0. A Poisson logistic regression model was created to assess both sexual activity or dysfunction, and in each model we included the variables that presented significance of association ( *p* < 0.05) or a trend to association ( *p* < 0.2) in the bivariate analysis: BMI, UI and fecal incontinence. The present study was approved by each local institutional ethics committee under registration number (CAAE 96636518.3.0000.5327).

## Results

In the present study, we analyzed 140 adult female SB patients, with a mean age of 27 (range: 18 to 42) years and a mean BMI of 26.2 (range: 18 to 43) Kg/m ^2^ , who were interviewed in Spain (89.3%) and Brazil (10.7%). Most patients were single (85%) women living with their parents/family (82.1%) who economically-dependent on them (66.4%). Their level of schooling was most commonly Elementary School (61.4%). Myelomeningocele at lower levels (91.4%) associated with hydrocephalus (82.9%) was the most common SB presentation at birth. Approximately 77.1% presented mobility without the need of aids, while 22.9% were wheelchair-dependent. In total, UI occurred in 83.6% of the patients, fecal incontinence was present in 64.3%, and 16.4% claimed a history of psychological disorder.

**Fig. 1:**
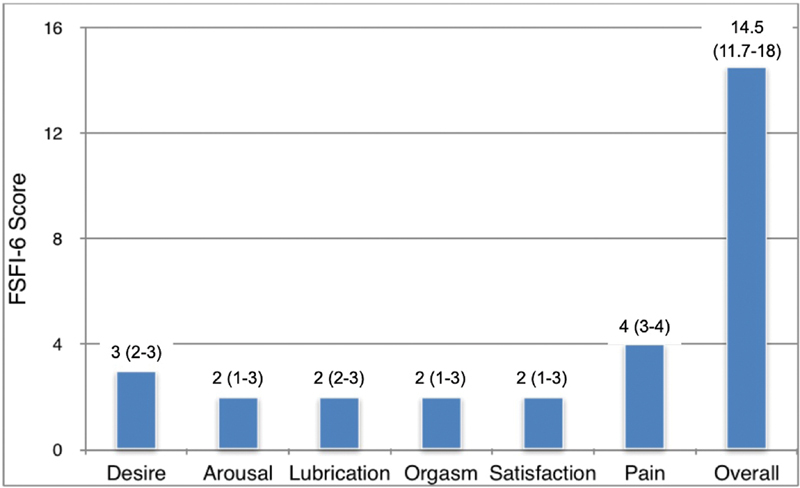
Median total score and scores for the domains of the six-item version of the Female Sexual Function Index (FSFI-6) among sexually-active women with spina bifida. Data are presented as medians (25th percentile––75th percentile).

Regular annual gynecological (GO) follow-up was a routine for 17.9% of the patients, irregular previous GO consultations occurred in 67.9%, and 14.3% had never had a single GO evaluation. Half of the patients had had sexual activity at least once in their life, and most (85%) were single women. Among those sexually active, most (57.1%) did not use any contraception method. Gestational history was present in 6 (4.3%) patients, all of them subitted to deliveries by cesarean section without complications ( Table 1 ). Sexual dysfunction was present in 84.3% of the sexually-active patients, with a median FSFI-6 total score of 14.5 (range: 4 to 26). The scores on specific domains of the FSFI-6 were also analyzed among the sexually-active women ( Fig. 1 ).

**Table 1:** Gynecological care and sexuality characteristics of spina bifida patients

Characteristic	n (%)
*Gynecological examination*	
Irregular visits	95(67.9)
Regular visits	25(17.9)
Has never undergone a gynecological examination	20(14.3)
*Sexual activity*	
No	70(50)
Yes	70(50)
*Contraceptive method* ^a,b^	
No	40(57.1)
Yes	30(42.9)
*Pregnancy*	
No	134(95.7)
Yes	6(4.3)
*Sexual dysfunction* ^a^	
No	11(15.7)
Yes	59(84.3)

Notes: ^a^ Assessed only among sexually-active women; ^b^ includes hormonal and non-hormonal contraceptives.

The clinical characteristics of the patients were compared with their sexual activity and the presence of sexual dysfunction (FSFI-6 overall score > 19). The type of SB, spinal cord level, hydrocephalus, use of wheelchair, psychological disorder, and fecal incontinence were not statistically associated with differences in the rates of sexual activity or dysfunction. Obesity (BMI ≥ 30) had a significant association with sexual dysfunction ( *p* = 0.004; Fisher exact test), but no differences regarding sexual-activity rates ( *p* = 0.572). The presence of UI was associated with significant lower rates of sexual activity (continent: 78.3% versus UI: 44%; *p* = 0.003; Chi-squared test) and higher rates of sexual dysfunction (continent: 50% versus UI: 96.2%; *p* < 0.001; Fisher exact test) ( Table 2 ).

**Table 2:** Clinical characteristics and sexual outcomes

	Sexual activity n = 140		Sexual dysfunction* n = 70	
	No	Yes	No	Yes
Body mass index (Kg/m ^2^ )				
≤ 20	4(50)	4(50)	3(75)	1(25%)
20–25	18(41.9)	25(58.1)	1(4)	24(96%)
25–30	35(52.2)	32 (47.8)	6(18.8)	26(81.2%)
≥ 30	13(59.1)	9(40.9)	1(11.1)	8(88.9)
*p-value*	0.572 ^a^		0.004 ^b^	
Myelomeningocele	64(50)	64 (50)	11(17.2)	53(82.8)
Meningocele/Others (includes spina bifida occulta)	6(50)	6(50)	0(0)	6(100)
*p-value*	1 ^a^		0.580 ^b^	
Spinal cord level				
Lumbar or lumbosacral	64(50)	64(50)	10(15.6)	54(84.4)
Thoracic or Thoracolumbar	6(50)	6(50)	1(16.7)	5(83.3)
*p-value*	1 ^a^		1 ^b^	
Hydrocephalus				
No	13(54.2)	11(45.8)	2(18.2)	9(81.8)
Yes	57(49.1)	59(50.9)	9(15.3)	50(84.7)
*p-value*	0.654 ^a^		1 ^b^	
Deambulation				
Deambulates	52(48.1)	56(51.9)	9(16.1)	47(83.9)
Wheelchair	18(56.3)	14(43.7)	2(14.3)	12(85.7)
*p-value*	0.421 ^a^		1 ^b^	
Urinary incontinence				
No	5(21.7)	18(78.3)	9(50)	9(50)
Yes	65(55.6)	52(44.4)	2(3.8)	50(96.2)
*p-value*	0.003 ^a^		< 0.001 ^b^	
Fecal incontinence				
No	23(46)	27(54)	7(25.9)	20(74.1)
Yes	47(52.2)	43(47.8)	4(9.3)	39(90.7)
*p-value*	0.480 ^a^		0.092 ^b^	
Psychological disorder				
No	59(50.4)	58(49.6)	8(13.8)	50(86.2)
Yes	11(47.8)	12(52.%)	3(25)	9(75)
*p-value*	0.820 ^a^		0.386 ^b^	

Notes: ^a^ Chi-squared test; ^b^ Fisher exact test; female sexual dysfunction was assessed only among sexually-active women.

A Poisson logistic regression model using BMI, UI and fecal incontinence was created to assess both sexual activity and dysfunction. The only clinical variable that demonstrated significance with lower sexual activity ( *p* = 0.006) and more sexual dysfunction ( *p* = 0.004) in the regression analysis was UI. Patients who suffered from UI presented a prevalence ratio of 1.46 (95% confidence interval [95%CI]: 1.21–1.76) of sexual dysfunction, and a prevalence ratio of 0.78 (95%CI: 0.67–0.9) of sexual activity.

The sexual-function domains were also analyzed quantitatively. Obesity, type of SB, and deambulation status did not influence the scores of any sexual function domain. Those without hydrocephalus had better scores only in the orgasm domain. Fecal incontinence and UI were significantly associated with lower scores in all domains, except for pain ( Table 3 ).

**Table 3:** Clinical characteristics and specific domains of the 6-item version of the Female Sexual Function Index (FSFI-6) among sexually-active female spina bifida patients. Data are presented as medians (25th percentile––75th percentile)

	Desire	Arousal	Lubrication	Orgasm	Satisfaction	Pain	Overall
**Type of lesion**							
Myelomeningocele	3(2–3)	2(1–3)	2(2–3)	2(1–3)	2(1–3)	4(3–4)	14.5(11.25–18)
Meningocele/Others (includes spina bifida occulta)	2(2–3)	2(1.75–3.25)	2(1.75–3.25)	2(1.75–3)	1.5(1–2.25)	4.5(3–5)	15.5(11.75–17.5)
p-value*	0.542	0.655	0.786	0.761	0.345	0.247	0.891
**Spinal cord level**							
Lumbar or lumbosacral	3(2–3)	2(1–3)	2(2–3)	2(1–3)	2(1–3)	4(3–4)	15(11.25–18)
Thoracic or thoracolumbar	1.5(1–4)	2(2–3.5)	2(1–4)	2(2–2.5)	1(1–3.5)	4(3.75–5)	14.3(11.75–20.75)
p-value*	0.322	0.349	0.761	0.573	0.430	0.221	0.908
**Hydrocephalus**							
No	2(1–3)	2(1–3)	2(2–3)	3(2–4)	1(1–2)	4(4–5)	15(12–18)
Yes	3(2–3)	2(1–3)	2(2–3)	1(1–3)	2(1–3)	4(3–4)	14(11–18)
p-value*	0.303	0.802	0.701	**0.015**	0.328	0.241	0.703
**Deambulation**							
Deambulates	2.5(2–3)	2(1–3)	2(2–3)	2(1.25–3)	2(1–3)	4(3–4)	15(11.25–18)
Wheelchair	3(2–4)	2(0.75–3)	2.5(1.5–3)	1(0.75–2.25)	2(1–3.25)	4(2.25–4)	13.5(11.5–16.25)
p-value*	0.067	0.843	0.524	0.136	0.062	0.232	0.534
**Urinary incontinence**							
No	3.5(3–4)	3(3–3.25)	4(3–4)	3(2–3)	3.5(3–4)	4(3–4)	19.5(18–23)
Yes	2(1.25–3)	2(1–2)	2(1.25–3)	2(1–3)	1.5(1–2)	4(3–4)	13(11–15.75)
p-value*	**< 0.001**	**< 0.001**	**< 0.001**	**0.037**	**< 0.001**	0.381	**< 0.001**
**Fecal incontinence**							
No	3(2–4)	3(2–3)	3(2–4)	3(2–3)	3(2–4)	4(3–4)	18(13–21)
Yes	2(1–3)	2(1–2)	2(2–3)	2(1–2)	2(1–2)	4(3–5)	13(11–16)
p-value*	**0.007**	**0.009**	**0.017**	**0.006**	**0.004**	0.065	**0.003**
**Psychological disorder**							
No	3(2–3)	2(1–3)	2(2–3)	2(1–3)	2(1–3)	4(3–4)	14.5(11.75–18)
Yes	3(2–3)	2(2–3)	2(2–3)	3(1.25–3)	2(1–3.75)	4(3–4.75)	15.5(11.5–21.75)
p-value*	0.802	0.374	0.866	0.194	0.903	0.993	0.547

Note: ^*^ Mann-Whitney non-parametric test.

## Discussion

Spina bifida is a complex group of anatomical changes characterized by impaired fusion of the vertebral arches in the first 28 days of the embryo, and it is considered the main neurological birth defect. 1 Traditionally considered a condition of the pediatric population, SB has undergone major changes due to better medical care, and, nowadays, most patients reach adulthood. 2 Thus, the increase in life expectancy leads to a rising importance of sexuality among this population.

A sexual activity rate of 50% among the SB patients in the present study is concordant with previous research 6 7 8 14 that reported rates of sexual activity among women with the same condition ranging from 32% to 68%. This demonstrates important differences compared with the general female population from the countries involved in the study – Brazil and Spain –, which presented sexual intercourse rates ranging from 83.6% to 85.3%. 15 16 Also, the prevalence of sexual dysfunction in the present study (84.3%), compared with a large Brazilian sample of non-SB women with dysfunction rates of 49%, highlights the need to improve sexual care in SB. 17 The weak median scores found in the overall and specific-domains of the FSFI-6 among our patients are similar to the scores found in previous studies that quantitatively assessed sexuality in a quantitative matter. 8 18 Lee et al. 18 found lower overall and specific-domain scores in the FSFI of SB patients when compared with non-SB women who also suffered from sexual dysfunction, showing that these neurologic patients demand more attention to their sexual life than regular patients. 18

To comprehend the sexuality of female patients, it is important to assess their GO aspects. Our study demonstrated that only 17.9% of the SB patients had regular annual GO follow-up, meanwhile almost 15% had never undergone a single GO evaluation. Also, the prevalence of contraceptive methods used by sexually-active SB patients was much inferior compared with non-neurogenic female sexually-active patients who attended the gynecology outpatient clinic of the Brazilian institution in the present study (SB patients: 42.9%; regular patients: 91.9%), revealing the risk of inadvertent pregnancy among the population with SB. 19 Other studies 7 9 have already demonstrated a lack of contraception in these patients, which is believed to be related to inadequate sex education and unfamiliarity with the available options. Other factors that may contribute are the high rates of latex allergy and comorbidities associated with SB that restrict the use of contraceptives (such as epilepsy and the use of anticonvulsants; and reduced mobility and thromboembolic events). 20 21 The physiological process of pregnancy and the effects of fetal growth can exacerbate the manifestations of SB, such as bone abnormalities (mainly in the spine and hips), which could restrict mobility, cause pain, make vaginal delivery difficult, and hinder epidural analgesia. 20 22 Spina bifida patients have been encouraged to perform vaginal deliveries and follow the obstetric indications for cesarean section respecting their orthopedic limitations (auch as narrow pelvis or severe scoliosis). 20 23 Despite these recommendations, SB patients are still most often submitted to cesarean sections when compared with the general population. 23 In the present study, in spite of the low rates of contraception, only 4.3% had a history of pregnancy, and there were no major complications during deliveries, which were all cesarean sections.

The clinical characteristics of SB and the sexual outcomes have been analyzed qualitatively and quantitatively. The UI status was the most relevant factor, since it impaired either sexual activity rates and worsened the overall and specific-domain scores on the FSFI-6. The only aspect that did not suffer significant influence from the UI was the pain domain. These findings are consonant with those of previous studies, including the specific data from Gamé et al., 6 who also observed that the desire, arousal and lubrication domains suffered negative effects from UI among SB patients. 6 7 8 Other non-neurogenic conditions that caused UI also showed that it has major impact in female sexual life, mainly due to the fear of unpredictable incontinence during sex. Urinary incontinence impairs the self-esteem and promotes anxiety, which could contribute to these findings. 24 25 Although fecal incontinence did not promote significant differences in the rates of sexual activity, we found that it influenced negatively, in a similar manner to that of UI, in all sexual-function domains but pain. Few studies 26 27 assessed fecal incontinence and sexuality, with a limited inference that it could impair the social life and sexual perception of the SB patients. 26 27 Neurological characteristics (type of SB, spinal cord level, hydrocephalus, walking ability, and the presence of concomitant psychological disorders) showed little influence in the sexual outcomes. The only significant finding is that those without hydrocephalus had better scores on the orgasm domain of the FSFI-6. Two previous studies 9 28 have described that SB patients with hydrocephalus demonstrated inferior sexual activity, fewer sexual partners, and more sexual dysfunction. The fact that hydrocephalus is caused by Arnold-Chiari type-2 cerebellar malformation could explain the orgasm interference, since the cerebellum demonstrated increased activity during orgasm in functional magnetic resonance imaging studies. 29

There are some limitations to the present study that should be considered potential bias. The instrument to evaluate sexual function (FSFI-6) was originally validated in women who attended outpatient clinics for reproductive medicine in Italy, and the Brazilian Portuguese version was assessed in middle-aged patients. There are no validated sexual questionaries specific for SB patients, in which is a limitation of the present study. Another limitation is that UI was simplified in yes or no groups, not taking into account the different types that could be present (sphincteric insufficiency, detrusor hyperactivity, or both), because it was not possible to access the videourodynamics exams from most of the sample.

## Conclusion

The clinical aspects of SB patients, such as UI and fecal incontinence, should be properly addressed by their doctors, since they are associated with reduced sexual activity and lower FSFI-6 scores in the overall or specific domains. There is also a need to improve GO care among sexually-active SB patients, since most do not use any contraceptive methods, and are at risk of inadvertent pregnancy.
